# Patient and dental practitioner acceptance of artificial intelligence in dental care: a cross-sectional study in Saudi Arabia's eastern province

**DOI:** 10.3389/froh.2026.1794097

**Published:** 2026-06-10

**Authors:** Shimaa Rifaat, Ahmad AlNassar, Anas AlQuraishi, Naif AlQahtani, Taiseer Wafai, Faraz Farooqi, Balgis Gaffar, Noha Taymour

**Affiliations:** 1 Department of Restorative Dental Sciences, College of Dentistry, Imam Abdulrahman Bin Faisal University, Dammam, Saudi Arabia; 2 College of Dentistry, Imam Abdulrahman Bin Faisal University, Dammam, Saudi Arabia; 3 Department of Dental Education, College of Dentistry, Imam Abdulrahman Bin Faisal University, Dammam, Saudi Arabia; 4 Department of Preventive Dental Sciences, College of Dentistry, Imam Abdulrahman bin Faisal University, Dammam, Saudi Arabia; 5 Department of Substitutive Dental Sciences, College of Dentistry, Imam Abdulrahman Bin Faisal University, Dammam, Saudi Arabia

**Keywords:** AI training, artificial intelligence, dentistry, digital health, innovation, patient satisfaction, Saudi arabia, trustworthiness

## Abstract

**Introduction:**

Artificial Intelligence (AI) is steadily emerging in dental health care field, yet successful implementation depends on stakeholder acceptance. Few studies have directly compared patient and dental practitioner perceptions within the same cultural and healthcare context.

**Objective:**

This study aimed to describe and compare awareness and acceptance of AI in dental care among patients and practitioners in Saudi Arabia's eastern province, and to explore associations with key demographic and professional characteristics identifying factors influencing its adoption.

**Methods:**

A cross-sectional self-completed questionnaire survey for patients and dental practitioners in the Eastern Province (Saudi Arabia) was conducted. Data was collected from patients and public communities who were willing to participate in the questionnaire. The final questionnaire was provided in English and Arabic versions. It was composed of 5 sections including 38 questions. The questions analyzed the participants’ demographic data, evaluation of technical affinity, awareness of AI usage, perception of different aspects of AI in dental healthcare, and concerns related to AI. The validated questionnaire assessed demographics, technical affinity, AI awareness, usage, perception, and concerns. Data were analyzed using descriptive statistics, Chi-square tests, Mann–Whitney U test, Kruskal–Wallis test, and correlation analysis.

**Results:**

Awareness of AI was remarkably high (>90%) across all demographics. AI usage was significantly higher among younger participants and males (*p* < 0.05). Patients expressed generally positive perceptions (mean scores 3.3–4.1) but strongly emphasized that dental practitioners must retain final diagnostic and treatment authority (mean = 4.0 ± 1.05). Among practitioners, formal AI training was significantly associated with higher perceived decision-making accuracy (*p* = 0.019), patient satisfaction (*p* = 0.017), and clinical outcomes (*p* = 0.012).

**Conclusion:**

This study reveals a positive but cautious attitude toward AI in dentistry, where patients prioritize data privacy and the human touch, while practitioners advocate for a “human-in-the-loop” model that preserves clinical authority. Formal AI training was associated with higher perceived scores among dental practitioners highlighting the potential value of educational initiatives in fostering AI adoption. Bridging this perception gap requires a holistic strategy integrating comprehensive ethical frameworks, targeted education, and a strong commitment to human-centered care.

## Graphical Abstract

**Figure:**
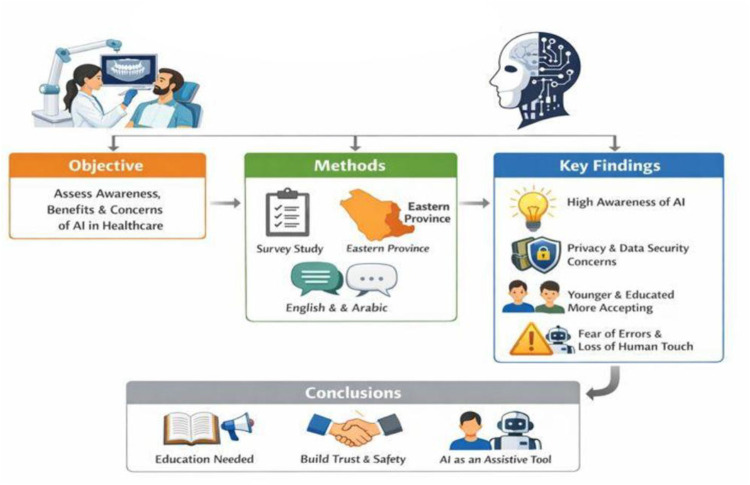

## Introduction

The landscape of modern dentistry is being fundamentally reshaped by artificial intelligence (AI) (1). It emerges from algorithmic interpretation of radiographs for caries detection (2) and automated cephalometric analysis in orthodontics (3) to AI-driven design of prosthetic restorations (4) and predictive models for periodontal disease progression (5), AI applications are rapidly transitioning from theoretical concepts to clinical tools (6). AI can assist dentists to make better clinical decisions or even might replace human judgement in certain functional areas of radiographic interpretation (7). This technological revolution is not confined to the clinic; it has fundamentally reshaped how patients engage with health information. In today's digitally connected world, individuals are no longer passive recipients of care but active information-seekers, turning to online platforms and social networks for guidance, community, and validation (8). This trend is further accelerated by the rise of advanced large language models (LLMs), which both patients and practitioners now use to obtain instant information on complex topics (9).

Concurrently, AI is being leveraged to extract useful information from large patient populations assisting in real-time health risk alerts and outcome predictions (10). The successful integration of these powerful technologies is ultimately contingent upon human acceptance (11). Previous studies indicate that patient willingness is nuanced and influenced by factors such as the perceived invasiveness of the procedure; for example, patients were less willing to undergo AI-assisted robotic gum surgery than non-invasive teeth whitening (12, 13). Interestingly, individual characteristics like innovativeness can predict readiness to use AI, whereas sociodemographic factors such as age, gender, and education may not be significant predictors in some contexts (14, 15). This underscores the critical importance of early patient and public involvement and engagement (PPIE). As Lammons et al. (16) concluded, involving patients in the development and implementation of AI is crucial not only to safeguard their interests but also to increase the likelihood of public acceptance and maximize the technology's positive impact on health outcomes. While a growing body of literature has explored clinician perspectives on AI in technologically advanced regions of North America and Europe (17, 18), there is a lack of data from the eastern province. Specifically, Saudi Arabia, as part of its ambitious Vision 2030 transformation agenda, is making substantial investments in digital health infrastructure (19). This rapid modernization creates an urgent need to understand the readiness of its population and dental workforce to embrace AI (20). Furthermore, the patient voice, a crucial stakeholder in any care model, remains underrepresented in the discourse on AI in dentistry (21). This study addresses this critical gap by providing a comprehensive assessment of AI acceptance from the dual perspectives of patients and dental practitioners within the Saudi Arabian eastern province. Therefore, this study aimed to evaluate the understanding, perception, and acceptance of AI in dental care among the population of the eastern province of Saudi Arabia. We seek to identify the key drivers and barriers to AI adoption, with a specific focus on the interplay between patient trust, practitioner attitudes, and the influence of formal training.

The null hypothesis posits that there is no significant difference in the levels of awareness, perception, and acceptance of AI-based dental care between patients and dental practitioners, and that sociodemographic characteristics (age, gender, education) among participants do not significantly influence these attitudes.

## Methodology

A cross-sectional self-completed questionnaire-based survey was conducted between August 2024 and February 2025 in the eastern province of Saudi Arabia. All the participants were briefed about the aim of the study, ensuring that their participation is purely voluntary. The survey responses were designed to be anonymous to protect the identity of the participants. Ethical approval was obtained from the Institutional Ethics & Review board of Imam Abdulrahman bin Faisal University (IRB-2024-02-325).

According to Perneger et al. (22), a sample size of 10 participants was considered adequate for pretesting and refinement of the survey instrument. The purpose of this phase was to evaluate clarity, comprehensibility, feasibility, and internal consistency of the questionnaire rather than to conduct hypothesis testing. The results of the pretest were analyzed for reliability using Cronbach's alpha, inter-item correlation, and corrected item total correlation. Questions with a corrected item total correlation below the acceptable threshold were removed to ensure proper evaluation of the subsections. Additional improvements in wording and clarity were made based on participant feedback. Approximately 80% of participants were able to complete the questionnaire within 15 min. The final questionnaire was provided in both English and Arabic versions.

### Sample characteristics and sample size

The survey was conducted on a google document with shared links and scanned QR codes spread anonymously. The sample comprised both patients (members of the public seeking or receiving care) and dental practitioners. Given the focus on dental applications, specific attention was paid to the responses from the dental practitioner subgroup (dentists, laboratory technicians). The inclusion criteria were (a) the capability to read and understand the information given in the questionnaire, (b) the willingness to participate in the survey, and (c) aged 18 years old and more. Exclusion criteria may include: (a) incomplete responses, (b) participants with less than 18 years old, and (c) participants that are not from or not located in the eastern province region.

The sample size for this cross-sectional survey was calculated assuming a 95% confidence interval, a 5% margin of error, and 80% statistical power. The sample size calculation was based on estimating proportions for the primary descriptive objectives of the study rather than testing specific hypotheses. Subgroup and comparative analyses were considered exploratory in nature and were not specifically powered. Based on these parameters, the minimum required sample size was estimated to be 385 participants. To account for potential non-response and incomplete questionnaires, a 15% increase was applied, yielding a target sample size of approximately 443 participants. A total of 444 participants were included in the final analysis. Recruitment continued until the predetermined target sample size was achieved. Only fully completed questionnaires were included in the final analysis, while incomplete responses were excluded during data screening. Because the survey was distributed anonymously without a predefined sampling list, the exact number of individuals who accessed the survey could not be determined; therefore, a formal response rate was not calculated. During data screening, incomplete questionnaires were excluded from the final analysis. Incompleteness was more frequently observed in later sections of the survey, suggesting potential survey fatigue. No clear demographic differences were observed between complete and incomplete responses.

Likert-scale responses were coded numerically from 1 to 5 (1 = very negative, 2 = negative, 3 = neutral, 4 = positive, 5 = very positive). Accordingly, higher mean scores indicate more positive perceptions. Although some items were framed in terms of frequency, all responses were collected using a standardized 5-point Likert scale to capture overall tendencies in perception or behavior, ensuring consistency across the questionnaire.

### Survey administration

The survey responses were designed to be anonymous to protect the identity of the participants. It is composed of 5 sections including 39 questions. Following the initial demographic and general AI awareness questions, the survey was branched into two distinct modules. Module A, completed exclusively by dental practitioners, included sections on perceived impact on clinical practice and professional training and Module B, completed exclusively by patients, included a section on “Patient's Fears and Concerns about AI involvement and engagement (PPIE)”. The structure of the resulting questionnaire is given in Table 1.

**Table 1:** Survey Framework for Evaluating Patient and Practitioners Awareness and Acceptance of AI in Dental healthcare.

Questions	Number
Demographic data (drop list) - Age (18–25, 25–35, 35–45, 45–55, 55–65, >65) - Sex (Male, Female, Other) - Marital Status (Single, married, widowed, divorced, in a relationship) - Nationality (All the middle east countries) - Country of residence (Saudi Arabia, East province) - Level of education (No, elementary school level, middle school level, high school level, technical education, Bachelor, Masters, PhD) - Current occupation (Student, not employed outside the home, non-employed, employed, self-employed, retired, job seeker) - Personal health (Poor, fair, good, very good, excellent) - Are you a healthcare practitioner? (Yes, no) - Are you a technical expert (IT, computers, electronics) (yes, no)	10
Evaluation of the participants’ technical affinity. (very negative, negative, neutral, positive, very positive) - Do you have a smart phone? (yes, no) - How often do you use the internet? - How often do you get familiar with new devices, new programs, and ew apps? - How often do you like working on electronic devices (phones, computers)?	4
Personal Awareness of usages of AI. (yes, no, not sure) - Did you hear about AI? - Do you use any AI apps in your personal life? - Do you use any AI apps in your professional life? - Do you see that AI is Beneficial in your life? - Do you trust AI? - Preferred language for AI healthcare interactions Arabic only English only Bilingual (Arabic and English) Other (specify)	6
Module A: Perception of different aspects of AI in dental healthcare. (very negative, negative, neutral, positive, very positive) - AI will reduce the workload of dental care practitioners. - The usage of AI is an important facility to overcome practitioners’ shortage in some areas. The dental practitioner should have the final diagnosis, treatment plan, and therapy for the patient. - AI based decision support systems by dental practitioners should be used for patient care only if scientifically proven. - I approve my data anonymously accessible for non-commercial research centers if this can improve dental healthcare in the future. - The use of AI will change the dental profession positively - If the patient has been harmed, the dental practitioner should be penalized for not following AI recommendations. - Dental practitioners lack more information about AI. - I trust the assessment of AI less than the dental practitioner assessment. - Testing of AI decision validity should be tested before using on human being bodies. - By using AI, there will be less errors in the future. - I think that the use of AI will bring more benefits to dental care in the future.	12
Module B: Patients’ fear of AI (very negative, negative, neutral, positive, very positive) - I fear that AI tools may be manipulated and misused by a bad third party - I am worried about the security of my data. - I am afraid of technical malfunction of AI than a wrong decision from dental practitioner. - The usage of AI may impair the dental practitioner-patient relationship. - If the AI decided that the patient has a low chance of survival, dental practitioners will not fight for patient survival as much as before. - The influence of AI on medical decisions and treatment scares me. - AI should be controlled for positive output.	7

Data was collected using Google Forms, which automatically compiled responses into a secure digital spreadsheet. No personally identifiable information was collected, ensuring participant anonymity throughout the data handling process. The resulting dataset was then manually reviewed, cleaned, and coded by two independent researchers to ensure accuracy and consistency before being exported for statistical analysis.

### Statistical analysis

Statistical analyses were performed using IBM SPSS Statistics (version 29, IBM, USA). Descriptive statistics were used to summarize the data, with categorical variables presented as frequencies and percentages, and ordinal variables derived from Likert-scale responses summarized using means and standard deviations (mean ± SD). Participants completed a structured questionnaire that included a common demographic section, followed by different sections based on healthcare practitioner status. Analyses were conducted according to the relevant questionnaire sections and participant groups. Associations between categorical variables (e.g., demographic characteristics and AI awareness, trust, and usage) were assessed using Chi-square tests (Tables 3, 4). Comparisons of Likert-scale scores between two independent groups were performed using the Mann–Whitney *U* test, while comparisons across more than two groups were conducted using the Kruskal–Wallis test (Tables 5–7). Relationships between perception, fear, and trust constructs were examined using Spearman's correlation coefficient. All statistical tests were selected based on the type and distribution of variables. Given the exploratory nature of the analyses and multiple comparisons performed, findings should be interpreted with caution. A *p*-value ≤0.05 was considered statistically significant.

## Results

### Demographic characteristics

The study cohort comprised 444 participants, categorized into dental care practitioners (17.8%; *n* = 79) and non-practitioners (82.2%; *n* = 365). The age distribution was diverse, with the largest proportion of respondents aged 18–25 years (34.9%), followed by the 36–45 (19.8%) and 26–35 (18.9%) age groups. The sex distribution was nearly equal, with males representing 51.4% and females 48.6% of the sample. Regarding marital status, 53.2% were married and 43.0% were single. Most respondents were Saudi nationals (90.5%) residing in Dammam (42.1%). In terms of educational attainment, 66.7% held a bachelor's degree, while 9.3% possessed a master's or doctoral degree. Among non-practitioners, the most frequent occupations were employed individuals (38.3%), students (27.7%), and housewives (14.2%).

Within the dental practitioner subgroup (*n* = 79), dentists constituted the largest proportion (46.8%), followed by dental administrators (21.5%), laboratory technicians (16.5%), and physicians (3.8%). Over half of the practitioners worked in government hospitals (51.9%), and 22.8% had more than 15 years of professional experience. Detailed demographic characteristics are presented in Table 2.

**Table 2:** Demographical characteristics of both participants .

Descriptive statistics		Frequency	Percent
Age	18–25	155	34.9
	26–35	84	18.9
	36–45	88	19.8
	46–55	75	16.9
	56–65	38	8.6
	>66	4	0.9
Sex	Male	228	51.4
	Female	216	48.6
Marital status	Single	191	43.0
	Married	236	53.2
	Widowed	4	0.9
	Divorced	11	2.5
	In a relationship	2	0.5
Nationality	Saudi	402	90.5
	Non-Saudi	42	9.5
Residence	Dammam	187	42.1
	Khobar	73	16.4
	Jubail	37	8.3
	Qatif	27	6.1
	Al Ahsa	9	2.0
	Other	111	25.0
Education Level	High school level	85	19.1
	technical education	22	5.0
	Bachelor	296	66.7
	Masters	35	7.9
	PhD	6	1.4
Profession	Student	123	27.7
	not employed outside the homehouse	63	14.2
	non-employed	11	2.5
	employed	170	38.3
	self-employed	14	3.2
	retired	47	10.6
	job seeker	16	3.6
Are you a health practitioner	Yes	79	17.8
	No	365	82.2
Professional role	Physician	3	3.8
	Dentist	37	46.8
	Nurse	1	1.3
	Laboratory Technician	4	5.1
	Pharmacist	13	16.5
	Allied Health Professional	4	5.1
	Dental Healthcare Administrator	17	21.5
Years of professional experience	Less than 2 years	28	35.4
	2–5 years	16	20.3
	6–10 years	11	13.9
	11–15 years	6	7.6
	more than 15 years	18	22.8
Type of healthcare facility:	Government Hospital	41	51.9
	Private Hospital	2	2.5
	Primary Dental Healthcare Center	1	1.3
	Specialized Medical Center	2	2.5
	Other	33	41.8

### AI awareness and trust among non-practitioners

Awareness of artificial intelligence was high across all groups, exceeding 90% in most categories (Table 3). For example, awareness reached 98.7% among participants with bachelor's degrees compared to 93.2% among those with high school education (*p* = 0.023). Trust in AI varied across groups but remained moderate overall. The highest proportion reporting trust was observed among younger participants aged 18–25 years (31.4%), while lower levels were observed in older age groups.

**Table 3:** Comparison of AI awareness and trust among the patients.

		Fear of AI				Trust AI			
Demographic Data	Demographic Characteristic	Yes	No	Not Sure	*P*-Value	Yes	No	Not Sure	*P*-Value
Age	18–25	99.2%	0.0%	0.8%	0.276	31.4%	17.8%	50.8%	0.276
	26–35	97.0%	0.0%	3.0%		42.4%	24.2%	33.3%	
	36–45	97.4%	1.3%	1.3%		36.8%	18.4%	44.7%	
	46–55	97.1%	1.5%	1.5%		38.2%	19.1%	42.6%	
	56–65	90.9%	0.0%	9.1%		24.2%	36.4%	39.4%	
	>66	100.0%	0.0%	0.0%		50.0%	0.0%	50.0%	
Sex	Male	97.3%	0.0%	2.7%	0.285	38.3%	18.1%	43.6%	0.308
	Female	97.2%	1.1%	1.7%		32.2%	23.7%	44.1%	
Education level	High school level	93.2%	0.0%	6.8%	0.023*	28.4%	21.6%	50.0%	0.575
	technical education	95.0%	5.0%	0.0%		50.0%	20.0%	30.0%	
	Bachelor	98.7%	0.4%	0.8%		35.6%	20.1%	44.4%	
	Masters	96.8%	0.0%	3.2%		38.7%	25.8%	35.5%	
	PhD	100.0%	0.0%	0.0%		100.0%	0.0%	0.0%	

* Statistically Significant at 0.05.

### AI usage patterns

AI usage differed across demographic groups (Table 4). Participants aged 18–25 years reported the highest usage, with 90.7% indicating personal use and 83.9% professional use. In contrast, usage declined with increasing age and was minimal among those older than 66 years. Males reported higher usage compared to females for both personal (72.3% vs. 54.2%) and professional (59.6% vs. 46.3%) purposes. Similarly, higher usage was observed among participants with higher education levels and those identifying as technical experts. A similar pattern was observed across education levels, where participants with bachelor's and postgraduate degrees reported higher usage compared to those with lower educational attainment.

**Table 4:** Comparison of usage and demographic status of participants.

		Use AI personally				Use AI professionally			
Demographic Data	AI usage Years	Yes	No	Not Sure	*P*-Value	Yes	No	Not Sure	*P*-Value
Age	18–25	90.7%	5.1%	4.2%	0.001*	83.9%	11.0%	5.1%	0.001*
	26–35	71.2%	25.8%	3.0%		56.1%	37.9%	6.1%	
	36–45	47.4%	34.2%	18.4%		38.2%	47.4%	14.5%	
	46–55	42.6%	47.1%	10.3%		33.8%	54.4%	11.8%	
	56–65	39.4%	39.4%	21.2%		18.2%	57.6%	24.2%	
	>66	0.0%	75.0%	25.0%		0.0%	75.0%	25.0%	
Sex	Male	72.3%	18.1%	9.6%	0.001*	59.6%	29.3%	11.2%	0.013*
	Female	54.2%	35.6%	10.2%		46.3%	44.1%	9.6%	
Education level	High school level	50.0%	37.8%	12.2%	0.104	37.8%	43.2%	18.9%	0.005*
	technical education	45.0%	40.0%	15.0%		30.0%	60.0%	10.0%	
	Bachelor	68.6%	23.0%	8.4%		59.0%	33.9%	7.1%	
	Masters	67.7%	19.4%	12.9%		58.1%	25.8%	16.1%	
	PhD	100.0%	0.0%	0.0%		100.0%	0.0%	0.0%	
Technical expert	Yes	73.0%	16.4%	10.7%	0.007*	66.4%	27.0%	6.6%	0.001*
	No	58.8%	31.7%	9.5%		46.5%	41.2%	12.3%	

* Statistically Significant at 0.05.

### Patient perception of AI

Perception scores ranged from 3.3 to 4.1 on a 5-point scale, reflecting generally positive attitudes toward AI in dental care (Table 5). The highest mean score was for the statement emphasizing that the dental practitioner should retain final responsibility for diagnosis and treatment (4.0 ± 1.05).

**Table 5:** Comparison of perception of AI among patients’.

Perception of AI	Age of participants							Gender		
	18–25y	26–35y	36–45y	46–55y	56–65y	>66y	*p*-value	Male	Female	*p*-values
Artificial intelligence will reduce the workload on dental healthcare practitioners.	3.47 ± 0.96	3.32 ± 0.91	3.51 ± 0.89	3.46 ± 0.82	3.33 ± 0.82	4	0.565	3.43 ± 0.92	3.46 ± 0.86	0.732
The use of artificial intelligence is an important means of overcoming the shortage of practitioners in some fields.	3.37 ± 1.05	3.24 ± 0.86	3.22 ± 1.04	3.13 ± 0.91	3.39 ± 0.79	3.75 ± 0.5	0.489	3.25 ± 1	3.31 ± 0.94	0.549
The healthcare practitioner must have a final diagnosis, treatment plan, and treatment plan for the patient before using AI.	4 ± 1.05	4.15 ± 0.88	3.99 ± 1.06	3.99 ± 0.68	3.79 ± 0.78	3.75 ± 0.5	0.588	3.88 ± 0.96	4.12 ± 0.9	0.013*
AI-based decision support systems should only be used by practitioners for patient care if scientifically proven.	3.78 ± 0.97	3.65 ± 0.85	3.67 ± 0.93	3.75 ± 0.74	3.97 ± 0.73	3.75 ± 0.5	0.603	3.71 ± 0.92	3.79 ± 0.83	0.397
I consent to my data being made anonymously available to non-commercial research centers if this would improve future healthcare.	3.57 ± 1.05	3.64 ± 0.95	3.66 ± 1.07	3.63 ± 1.01	3.48 ± 1.18	3.5 ± 0.58	0.967	3.51 ± 1.06	3.7 ± 1	0.079
The use of artificial intelligence will 4ly change the medical profession.	3.6 ± 0.85	3.39 ± 1.14	3.49 ± 0.97	3.34 ± 0.94	3.67 ± 0.74	3.25 ± 0.5	0.353	3.54 ± 0.98	3.44 ± 0.9	0.301
If a patient is harmed, the practitioner should be penalized for not following AI recommendations.	2.78 ± 1.04	2.86 ± 1.12	2.96 ± 1	2.82 ± 0.98	2.76 ± 0.71	3.25 ± 0.5	0.784	2.85 ± 1.06	2.84 ± 0.94	0.887
Healthcare practitioners may lack more information about AI.	3.45±±0.97	3.3 ± 0.98	3.28 ± 0.83	3.31 ± 0.82	3.27 ± 0.67	3.5 ± 0.58	0.751	3.31 ± 0.94	3.38 ± 0.83	0.487
I trust AI assessment less than practitioner assessment.	3.22 ± 1.15	3.15 ± 1.18	2.93 ± 1.01	2.96 ± 0.89	3.18 ± 0.81	3.25 ± 0.5	0.399	3.06 ± 1.07	3.14 ± 1.03	0.484
AI decisions must be tested before they are used on human bodies.	4.37 ± 0.88	3.95 ± 1.21	4 ± 1.18	4.16 ± 0.87	4.09 ± 0.8	3.75 ± 0.5	0.058	4.19 ± 1.01	4.11 ± 1.01	0.457
With artificial intelligence, there will be fewer errors in the future.	3.42 ± 0.95	3.39 ± 0.89	3.42 ± 1.01	3.6 ± 0.92	3.64 ± 0.7	3.75 ± 0.5	0.56	3.57 ± 0.91	3.37 ± 0.92	0.036*
I believe the use of artificial intelligence will lead to more benefits for healthcare in the future.	3.75 ± 0.92	3.68 ± 0.93	3.66 ± 1.01	3.81 ± 0.78	3.76 ± 0.66	4.25 ± 0.96	0.757	3.79 ± 0.89	3.68 ± 0.91	0.271

* Statistically Significant at 0.05.

In contrast, lower agreement was observed for penalizing dental practitioners for not following AI recommendations (2.8 ± 1.0). Some variation by sex was noted, with females reporting higher agreement regarding practitioner responsibility, while males reported slightly higher agreement that AI may reduce errors.

### Fear and concerns regarding AI

Across educational and gender groups, fear scores were moderate, ranging from 3 to 3.6 on a 5-point scale (Table 6). The highest mean scores were specifically for worries regarding data misuse (3.63 ± 1.06) and technical malfunction (3.73 ± 1.08). A statistically significant difference was observed for the statement “AI must be controlled to achieve positive results” (*p* = 0.048), differences by sex were minimal.

**Table 6:** Fear and concerns about AI.

Perception of AI	Education					Gender		
	High school level	technical education	Bachelor	Masters	*p*-value	Male	Female	*p*-values
I fear that AI tools will be manipulated and misused by bad third parties.	3.28 ± 1.14	3.3 ± 0.92	3.63 ± 1.06	3.48 ± 0.93	0.125	3.47 ± 1.02	3.59 ± 1.11	0.285
I am concerned about the security of my data.	3.41 ± 0.99	3.4 ± 0.82	3.49 ± 1.05	3.58 ± 0.99	0.891	3.41 ± 0.96	3.55 ± 1.07	0.177
I fear a technical glitch in AI more than a bad decision by a healthcare practitioner.	3.42 ± 1.11	3.55 ± 1	3.73 ± 1.08	3.32 ± 1.01	0.101	3.55 ± 1.06	3.69 ± 1.1	0.229
The use of artificial intelligence may impact the practitioner-patient relationship.	3.28 ± 1.08	3.25 ± 0.97	3.47 ± 1.01	3.32 ± 0.91	0.568	3.41 ± 0.91	3.41 ± 1.11	0.979
If AI decides that a patient has little chance of survival, practitioners will not struggle for the patient's survival as before.	2.93 ± 1.21	3.3 ± 1.03	2.93 ± 1.35	3.19 ± 1.08	0.546	3.03 ± 1.3	2.92 ± 1.27	0.409
I'm scared of the impact of artificial intelligence on medical decisions and treatment.	3.27 ± 1	3.25 ± 0.91	3.47 ± 1.11	3.13 ± 1.15	0.355	3.32 ± 1.06	3.46 ± 1.11	0.223
Artificial intelligence must be controlled to achieve 4 results.	4.03 ± 0.86	3.45 ± 1	4.06 ± 0.92	3.97 ± 0.87	0.048	3.98 ± 0.96	4.05 ± 0.86	0.486

### Correlation analysis

**Figure 1:**
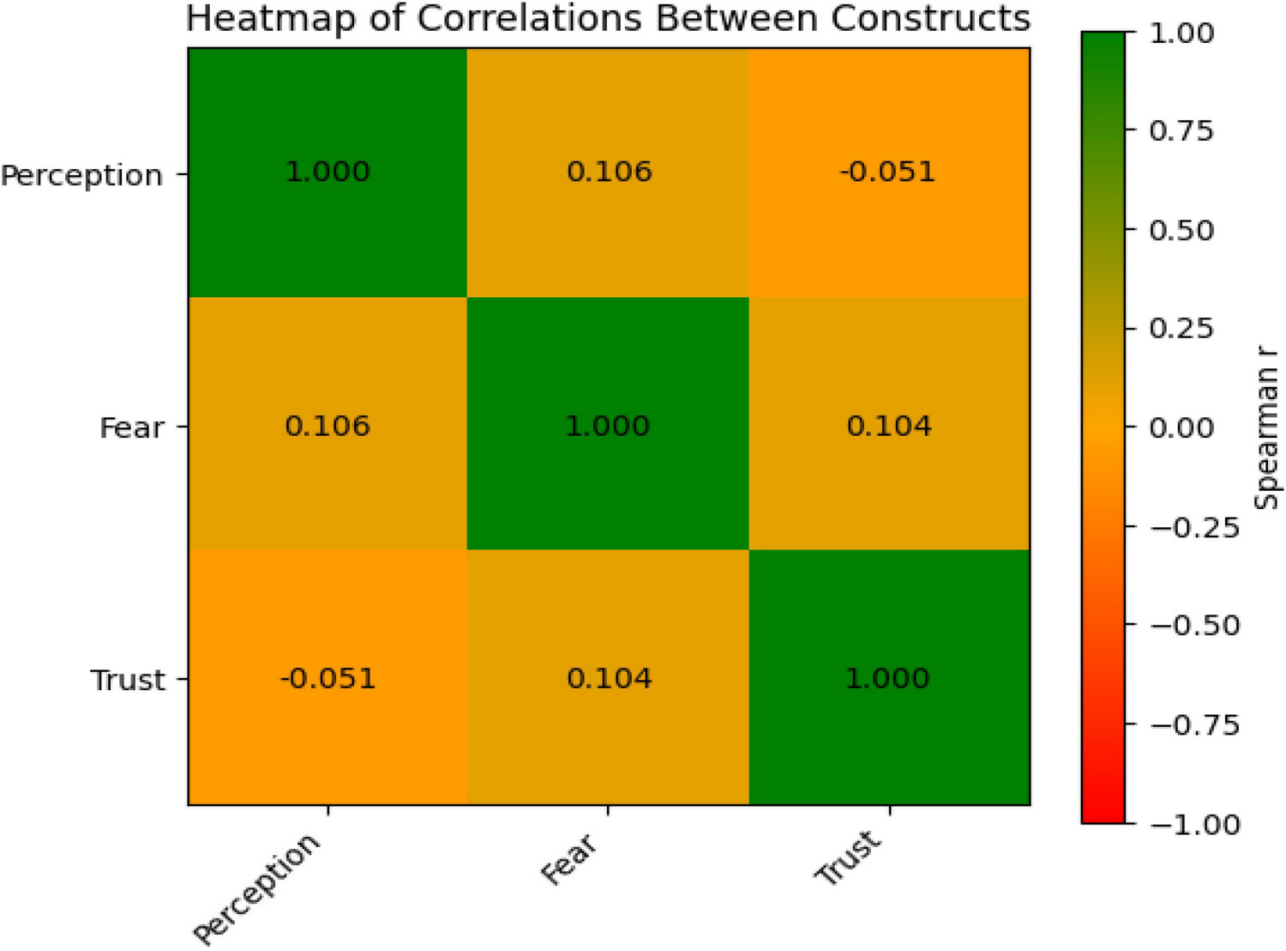
Heatmap of spearman correlation coefficients between perception, fear, and trust constructs.

Correlation analysis revealed a weak and positive relationship between perception and fear (*r* = 0.106, *p* < 0.05) and between fear and trust (*r* = 0.104, *p* < 0.05). In contrast, the correlation between perception and trust was very weak and not statistically significant (*r* = –0.051, *p* > 0.05). As shown in Figure 1, the heatmap illustrates the distribution of correlations between perception, fear, and trust constructs. The observed correlation coefficients were small in magnitude (*r* ≈ 0.10), indicating minimal relationships between variables.

### Perceived usefulness among dental practitioners

**Figure 2:**
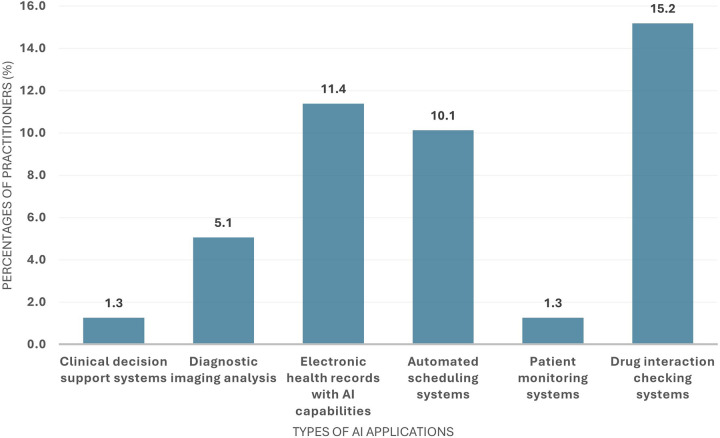
AI applications used by dental healthcare practitioners.

Perceived usefulness scores ranged from 3.1 to 3.8 across domains (Table 7). Higher scores were observed for research and data analysis (4.0 ± 0.63) and resource management (3.8 ± 0.98). Practitioners with 11–15 years of experience tended to report slightly higher usefulness scores, although differences across experience groups were limited. As shown in Figure 2, the most commonly reported AI application among dental practitioners was drug interaction checking systems (15.2%), followed by electronic health records with AI capabilities (11.4%) and automated scheduling systems (10.1%). Lower usage was observed for diagnostic imaging analysis (5.1%), while clinical decision support systems and patient monitoring systems were least reported (both 1.3%).

**Table 7:** Perceived usefulness among the dental practitioners.

	Professional experience					
Questionnaire item	Less than 2 years	2–5 years	6–10 years	11–15 years	more than 15 years	*P*-values
Assess your current knowledge of AI in dental healthcare.	2.21 ± 0.83	2.19 ± 0.91	2.45 ± 0.93	3 ± 1.41	2.67 ± 1.14	0.268
Evaluate the usefulness of AI in the following areas: Diagnosis accuracy	3.11 ± 0.96	3.25 ± 0.58	3.18 ± 0.98	3.5 ± 0.84	3.44 ± 0.78	0.676
Evaluate the usefulness of AI in the following areas: Treatment planning	3.21 ± 0.83	3.31 ± 0.48	3.45 ± 0.93	3.33 ± 1.37	3.33 ± 0.84	0.95
Evaluate the usefulness of AI in the following areas: Patient monitoring	2.93 ± 1.02	3.5 ± 0.63	3.36 ± 0.92	3.67 ± 1.03	3.28 ± 0.83	0.187
Evaluate the usefulness of AI in the following areas: Administrative tasks	3.29 ± 0.98	3.44 ± 0.89	3.55 ± 0.82	3.83 ± 0.98	3.44 ± 0.98	0.749
Evaluate the usefulness of AI in the following areas: research and data analysis	3.46 ± 0.88	4 ± 0.63	3.55 ± 0.69	4 ± 0.63	3.83 ± 0.51	0.105
Evaluate the usefulness of artificial intelligence in the following areas: Resource management	3.29 ± 1.01	3.38 ± 0.62	3.73 ± 0.65	3.83 ± 0.98	3.44 ± 0.92	0.507
Evaluate the usefulness of AI in the following areas: Clinical documentation	3.25 ± 0.93	3.31 ± 0.7	3.82 ± 0.6	3.5 ± 1.22	3.17 ± 1.04	0.384
Evaluate the usefulness of AI in the following areas: Patient engagement	3 ± 0.98	3.19 ± 0.66	3.18 ± 0.75	3.5 ± 1.22	3.39 ± 0.85	0.571

Table 7 presents perceived usefulness among healthcare practitioners. Healthcare professionals reported moderate to high perceptions of AI usefulness across multiple domains (mean range 3.1–3.8). The highest scores were for research and data analysis (mean = 4.0 ± 0.63 among 2–5 year and 11–15-year experience groups) and resource management (3.8 ± 0.98). Differences across experience levels were not statistically significant (*p* > 0.05), although practitioners with 11–15 years of experience consistently rated usefulness higher. Usefulness ratings for diagnosis accuracy, treatment planning, and patient monitoring were all above 3.0.

### Perceived differences in clinical and professional outcomes to formal AI training

**Figure 3:**
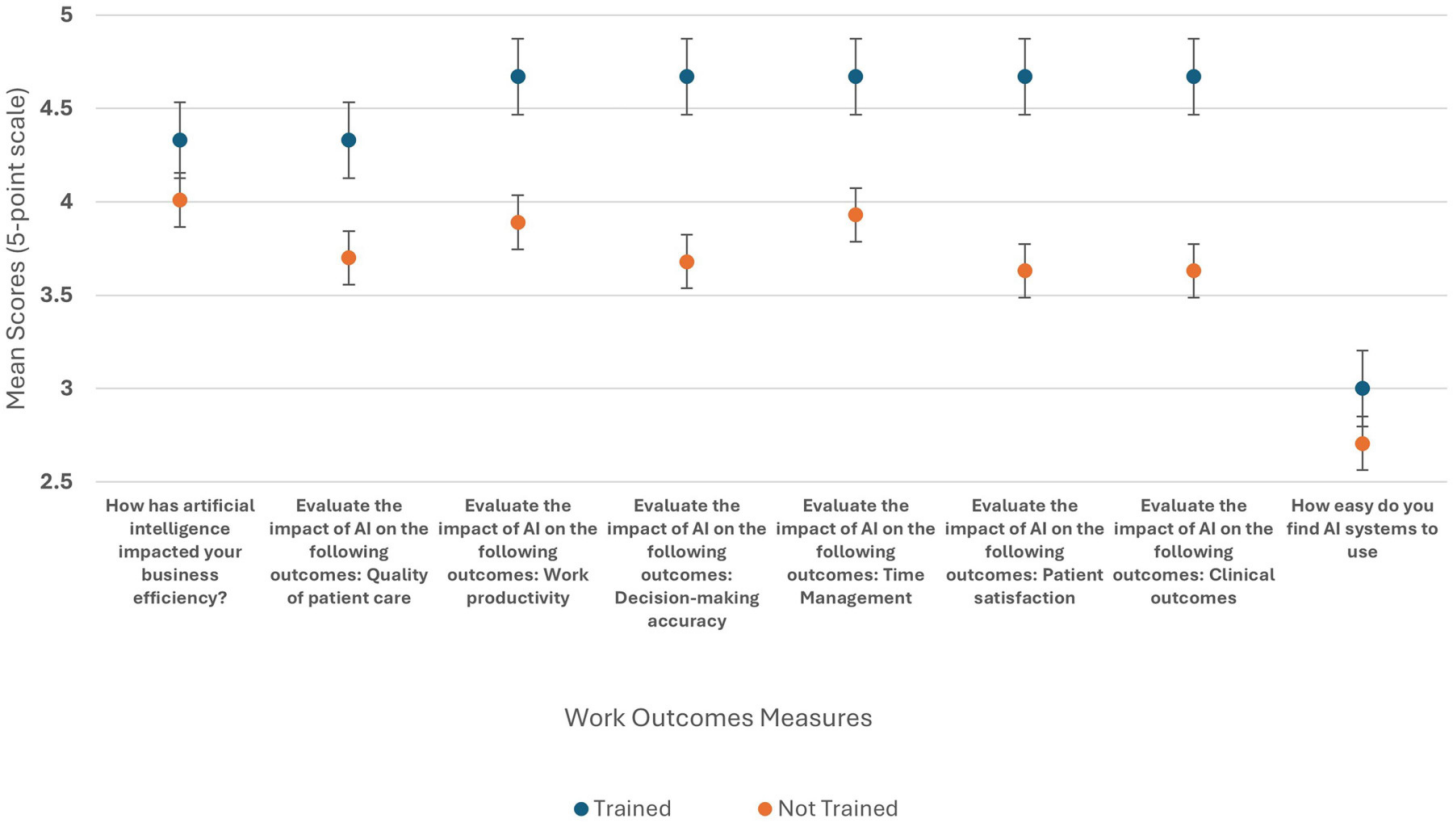
Impact of AI on work outcomes among dental health care practitioners.

Differences in work outcomes according to formal AI training are presented in Figure 3. As shown in Figure 3, dental practitioners with formal AI training reported higher mean scores for patient satisfaction (4.67 ± 0.58 vs. 3.68 ± 0.70) and perceived clinical outcomes (4.67 ± 0.58 vs. 3.63 ± 0.69). Higher scores were also observed for decision-making accuracy in the trained group (4.67 ± 0.58 vs. 3.63 ± 0.73), although differences for several outcomes, including productivity and efficiency, were small.

### Trust and reliability among dental practitioners

**Figure 4:**
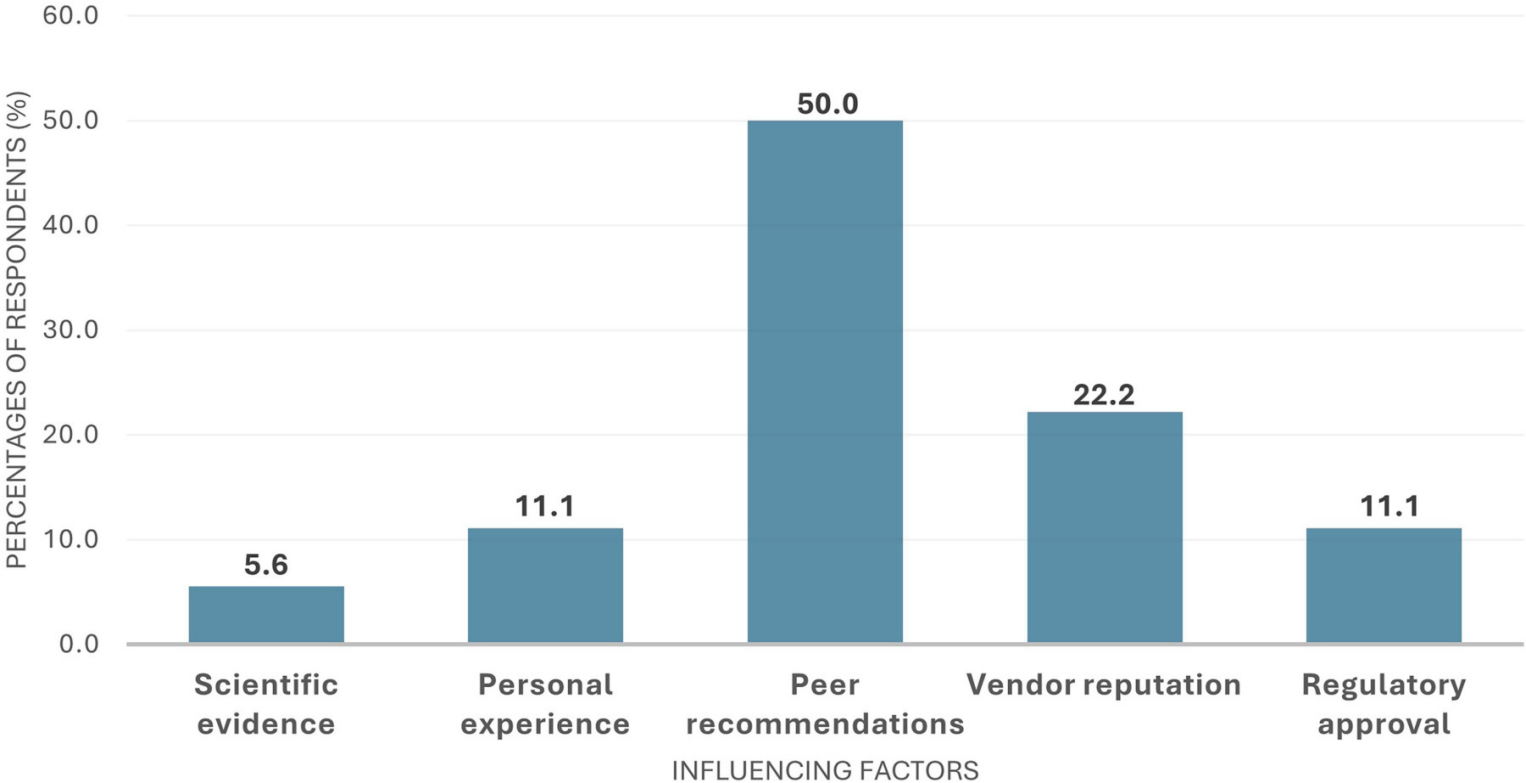
Factors that influence trust in AI.

Trust scores ranged from 2.8 to 4.0 across domains (Table 8). Dental practitioners with AI training reported higher trust in diagnostic suggestions (4.0 vs. 2.8), treatment recommendations (3.33 vs. 2.95), and clinical decision support (3.67 vs. 2.96), although differences were modest. Figure 4 shows that peer recommendations were the most influential factor in shaping trust in AI (50.0%), followed by vendor reputation (22.2%). Other factors, including regulatory approval (11.1%), personal experience (11.1%), and scientific evidence (5.6%), were reported less frequently.

**Table 8:** Trust & reliability among the healthcare practitioners.

	Have you received any formal training in AI healthcare applications?		
Questionnaire item	Yes	No	*p*-values
Rate your trust in AI systems in terms of: Diagnostic suggestions	4±	2.8 ± 0.9	0.19
Rate your trust in AI systems in terms of: Treatment recommendations	3.33 ± 0.58	2.95 ± 0.81	0.42
Rate your trust in AI systems in terms of: Patient monitoring	3.67 ± 0.58	2.96 ± 0.93	0.197
Rate your trust in AI systems in terms of: Administrative tasks	3.67 ± 0.58	3.18 ± 0.95	0.386
Rate your confidence in AI systems in terms of: Clinical decision support	3.67 ± 0.58	2.96 ± 0.89	0.177
Rate your trust in AI systems in terms of: Risk prediction	3.67 ± 0.58	3.07 ± 0.96	0.285

Table 8 presents trust and reliability among healthcare practitioners. Overall trust in AI systems ranged between 2.8 and 4.0 on the 5-point scale. Practitioners with AI training reported higher trust means for diagnostic suggestions (4.0 vs. 2.8), treatment recommendations (3.33 vs. 2.95), and clinical decision support (3.67 vs. 2.96), although differences were not statistically significant (*p* > 0.05).

### Organizational support for AI implementation

**Figure 5:**
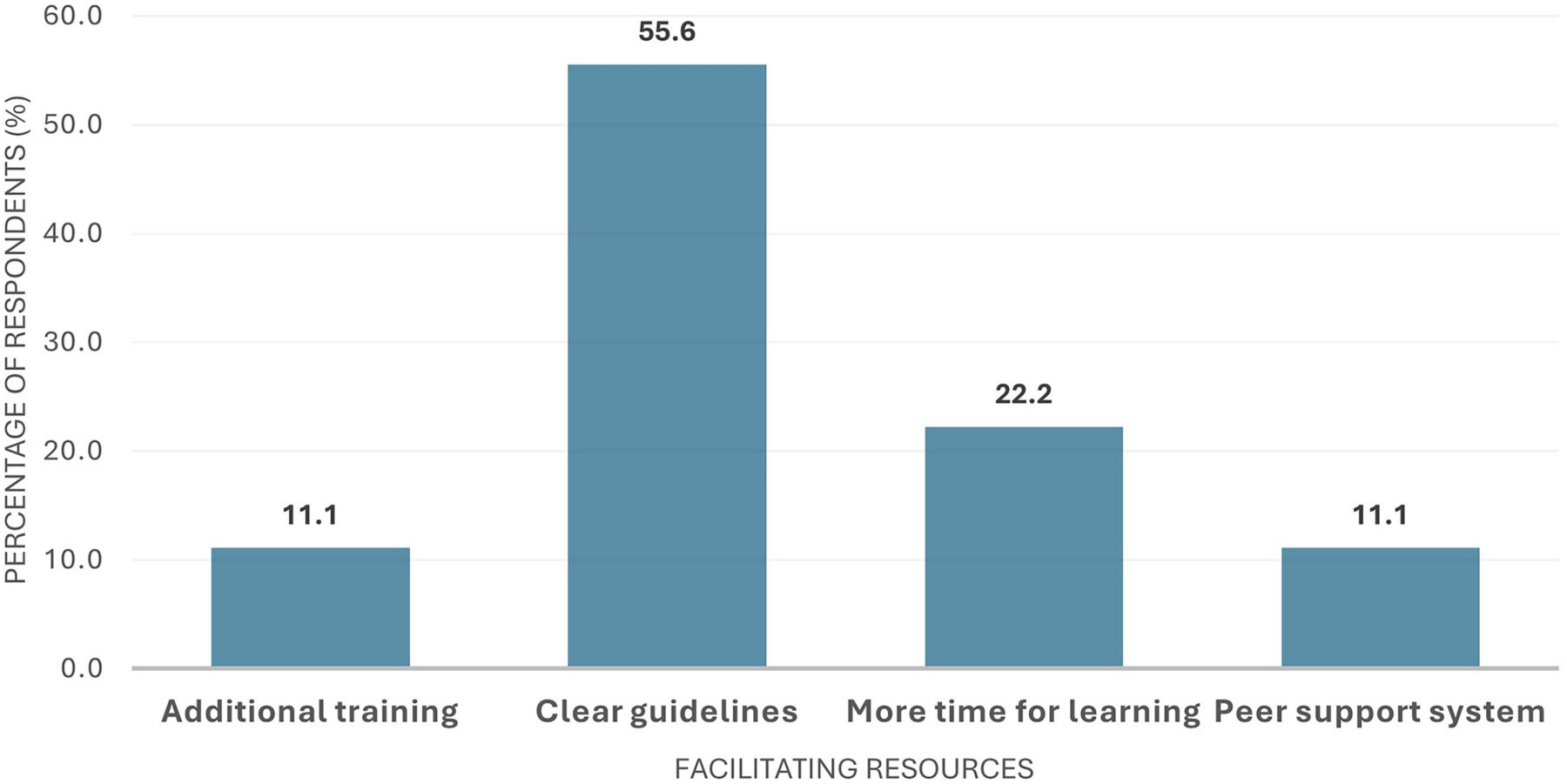
Resources that help to improve AI adoption.

Perceptions of institutional readiness for AI implementation were modest across all domains, with mean scores ranging from 2.7 to 3.1 (Table 9). No significant differences were observed between staff in government hospitals and those in private or specialized facilities regarding training provision, resource availability, or leadership engagement (*p* > 0.05). The highest mean scores were recorded for “clear policies and procedures” (3.08 ± 0.94) and “staff engagement initiatives” (3.11 ± 0.95), suggesting partial but insufficient administrative support. As illustrated in Figure 5, clear guidelines were the most frequently identified resource to support AI adoption (55.6%), followed by the need for additional time for learning (22.2%). Other factors, including additional training and peer support systems, were reported less frequently (both 11.1%).

**Table 9:** Assess your organization's support for AI implementation.

Assess your organization's support for AI implementation	Government Hospital	Private or other Hospital	*p*-values
Adequate training provided	2.73 ± 1.12	2.84 ± 1	0.646
Assess your organization's support for AI implementation: Clear policies and procedures	2.83 ± 1.12	3.08 ± 0.94	0.288
Availability of resources	2.8 ± 1.21	2.87 ± 1.12	0.809
Leadership support	2.71 ± 1.23	3.03 ± 0.97	0.207
For engagement initiatives	2.78 ± 1.15	3.11 ± 0.95	0.178

Table 9 presented organizational support for AI implementation. Perceptions of institutional readiness were modest across all domains (mean range 2.7–3.1 on a 5-point scale). Government hospital staff and those in private or specialized facilities did not differ significantly regarding training provision, resource availability, or leadership engagement (*p* > 0.05). The highest mean scores were observed for “clear policies and procedures” (3.08 ± 0.94) and “staff engagement initiatives” (3.11 ± 0.95), suggesting partial but insufficient administrative support.

### Professional outcome by role

**Figure 6:**
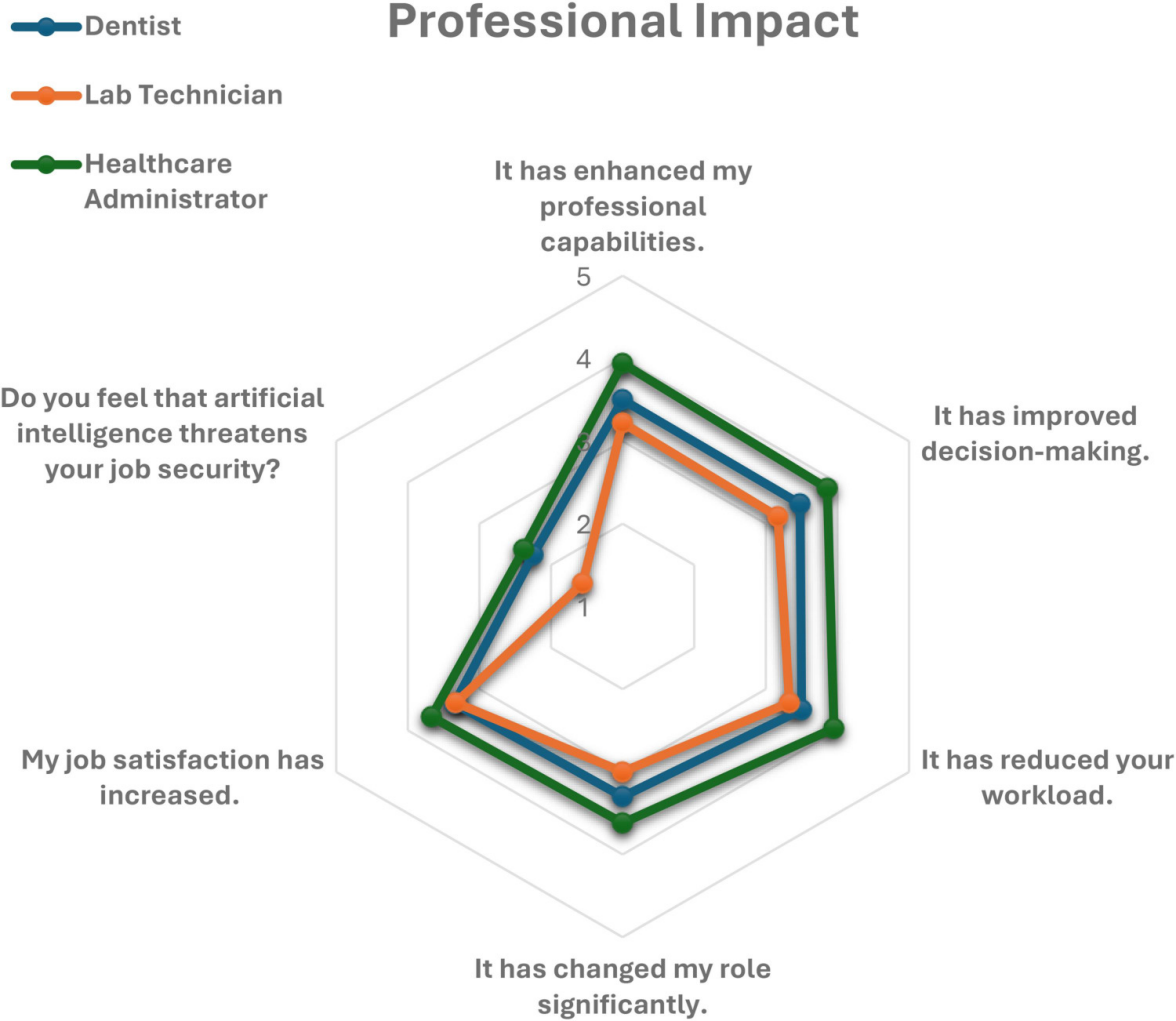
Professional impact of artificial intelligence across healthcare roles.

Dental administrators reported different outcomes compared to other roles (Figure 6). As shown in Figure 6, dental administrators reported higher scores for decision-making (3.86 ± 0.65) compared to other roles. They also showed higher mean scores for professional capabilities (3.94 ± 0.90) and lower perceived workload (3.95 ± 0.67). Concerns regarding job security were low across all groups (mean = 2.2), with minimal variation between roles.

## Discussion

This study explored patients' and dental practitioners' acceptance of artificial intelligence (AI) involvement in dental care in Saudi Arabia's eastern province. Our investigation revealed several key findings leading to rejection of the study hypothesis. First, awareness of AI among dental participants was remarkably high across all demographic groups (≥90%), with statistically significant differences by education level, where those with bachelor's degrees showed higher awareness (98.7%) compared to high school-educated participants (93.2%). The high awareness of AI across all demographic groups aligns with the increasing digitalization of dental healthcare in Saudi Arabia, consistent with the nation's Vision 2030 transformation goals (23). Aldogiher and Halim (23) provided critical evidence that customer engagement in digital health transformation is a cornerstone of the strategic change envisioned for the Kingdom. Our findings regarding the “General Public” cohort, particularly the high usage rates among younger, digitally native participants, reflect this broader national trend where residents are transitioning from passive recipients of care to active, engaged participants in the digital health ecosystem. This heightened engagement, fostered by national initiatives, likely explains why awareness levels in the eastern province are comparable to, or even exceed, those recently reported in other technologically advanced global regions.

The significant age-related disparity in AI usage, with the youngest cohort (18–25 years) reporting 90.7% personal use vs. 0% in the (>66 years) group, mirrors established research on technology adoption. Studies on electronic health record (EHR) and telehealth adoption have consistently shown that younger clinicians and patients are more likely to embrace new technologies (24). This divergence is often explained by differences in digital nativity, perceived ease of use, and technological self-efficacy. Our finding that males were more frequent AI users than females (72.3% vs. 54.2% personally) also find resonance in some literature, particularly from regions with more traditional gender roles, where men may have greater access to or encouragement for engaging with technology (25). However, this contrasts with studies from more gender-equal societies where such gaps are narrowing or non-existent, suggesting that cultural factors significantly mediate this relationship (26, 27). Third, while perceptions of AI were generally positive (3.3–4.1 on a 5-point scale), participants strongly emphasized the importance of human dental practitioners maintaining final diagnostic and treatment authority (mean = 4.0 ± 1.05).

The present study indicates a preference for augmented intelligence rather than autonomous AI systems. This finding aligns with the “human-in-the-loop” approach advocated by many AI ethicists, where AI serves as a decision support tool rather than an autonomous decision-maker (28). Patients and dentists worldwide consistently express a preference for augmented intelligence over autonomous AI (24, 29). Clinicians generally recognize AI's potential to improve efficiency, particularly in administrative tasks like documentation, but remain deeply concerned about the risks of ceding clinical judgment to machines. While AI is increasingly viewed as a “co-pilot” or supportive tool, significant apprehension exists regarding its reliability, the potential for deskilling of clinicians, and the risk of algorithmic bias (30, 31). Similarly, other studies exploring patient perspectives found that the “human touch,” empathy, and accountability of physicians were irreplaceable and seen as essential for building trust (32, 33).

The gender difference we observed for this item, with females scoring significantly higher (*p* = 0.013), is a novel contribution. While not extensively explored in prior literature, this may reflect different risk perceptions or a greater emphasis on relational aspects of care among female patients. The strong agreement across all groups that “AI decisions must be tested before they are used on human bodies” (mean > 4.0) further reinforces a cautious, evidence-based approach to integration, aligning with ethical frameworks proposed by organizations like the WHO (2021), which emphasize rigorous validation and human oversight as core principles for ethical AI in health (34, 35). Lastly, moderate concerns were identified regarding potential misuse of [AI by malicious parties and technical malfunctions which reflect rational apprehensions about emerging technologies. These concerns align with the Technology Acceptance Model, which identifies perceived risks as important factors in technology adoption (36).

The moderate levels of fear and concern, particularly regarding data misuse (mean = 3.63) and technical malfunction (mean = 3.73), which reflect rational apprehensions about emerging technologies. These concerns align with the Technology Acceptance Model, which identifies perceived risks as important factors in adoption of technology (37). Our findings showed that these fears were not significantly different by gender but were significantly associated with the belief that “AI must be controlled” among bachelor's degree holders (*p* = 0.048) offers. It suggests that higher education may not necessarily reduce fear but rather encourage a more sophisticated understanding of the technology's dual-use potential and the necessity for robust governance. This aligns with the concept of “informed trust,” where awareness of risks leads to calls for stronger safeguards rather than blind rejection (38).

Among dental practitioners, the perceived high usefulness of AI in research and data analysis (mean = 4.0) corroborates findings from previous studies in different medical fields (39, 40). AI's capacity to process vast datasets far exceeds human capability, making it a natural fit for research, pattern recognition, and administrative tasks (41).

Dental practitioners with prior AI formal training reported better perceptions of decision-making accuracy (4.67 vs. 3.68), patient satisfaction (4.67 vs. 3.63), and clinical outcomes (4.67 vs. 3.63). This finding should not be interpreted as an actionable result. Because this study was not designed to test the efficacy of training and utilized an exploratory, cross-sectional design without multivariable modeling, it only demonstrates a statistical association. This observation suggests that education may correlate with acceptance, but further longitudinal research is required to establish any causal impact. Previous studies demonstrated that targeted machine learning education for dentists significantly improved their confidence in and willingness to use AI-based clinical decision support tools (42, 43).

Conversely, the modest perceptions of organizational support (mean range: 2.7–3.1) and the lack of difference between government and private facilities highlight a critical implementation gap. This suggests that while individual dental practitioners may see the value of AI, the institutional ecosystem, lacking in training, resources, and clear policies, is not yet prepared to support its integration. This organizational inertia is a well-documented barrier to health technology innovation and suggests that top-down policy changes are needed to align institutional readiness with dental practitioner willingness (44, 45).

The outcomes of this study indicate that successful AI integration in dentistry necessitates a comprehensive, multi-stakeholder strategy spanning policy, clinical practice, and workforce development. At the patient and public level, targeted education campaigns using culturally adapted materials are crucial for building awareness and trust. Concurrently, continuous professional development must equip clinicians with AI literacy, empowering them to act as informed intermediaries between technology and patients. These efforts must be supported by regulatory and ethical frameworks that strengthen data protection, mandate human oversight, and ensure accountability. Ultimately, AI implementation must be human-centered, respecting cultural values and the clinician-patient relationship, while policymakers must ensure equitable access to prevent digital exclusion and align with national health agendas such as Vision 2030.This study adds to the limited body of evidence examining AI acceptance among patients in the eastern province in Saudi Arabia, a region where cultural, linguistic, and ethical contexts strongly shape dental healthcare behavior. While many studies focus on urban centers, this work provides valuable insights into the eastern province, a critical industrial and dental healthcare hub. The inclusion of specific “fear” constructs allows for a more nuanced understanding of the psychological barriers to AI adoption, moving beyond binary “accept/reject” metrics found in earlier literature. Furthermore, the quantitative demonstration of the “training effect” on work outcomes provides strong evidence base for educational investments. However, the study's focus on the eastern province may restrict generalizability to other Saudi regions or gulf populations with differing digital literacy levels. Self-reported measures may also introduce social desirability bias. In addition, due to the open-access recruitment strategy (QR codes and shared survey links), a formal response rate could not be calculated. This approach may introduce selection bias, as individuals with higher digital literacy or greater interest in technology may have been more likely to participate. Consequently, certain population groups, particularly those with limited digital access or lower familiarity with AI, may be underrepresented. These factors should be considered when interpreting the findings and their generalizability. Although this study provides valuable insight into patients' and dentists' acceptance of AI in dentistry, the findings should be interpreted to the local healthcare systems and digital infrastructure. Variability in primary dental healthcare organizations, technological readiness, and AI implementation across middle eastern cities may limit direct generalization. Therefore, the results may be more applicable to regions with comparable healthcare delivery models and academic environments, highlighting the need for future multicenter studies across diverse settings.

Future research should employ mixed-method and longitudinal designs to assess evolving perceptions following real-world AI deployment. Qualitative exploration of patient narratives, particularly concerning data privacy, autonomy, and interpersonal trust, would deepen understanding of cultural influences on AI acceptance. Additionally, intervention studies evaluating the impact of targeted educational or communication strategies could inform best practices for public engagement and policy formulation.

## Conclusion

This cross-sectional study demonstrates that while awareness of artificial intelligence in dental care is near-universal (>90%) among both patients and dental practitioners in Saudi Arabia's eastern province, yet significant usage disparities persist across age and gender groups. Patients strongly endorse a “human-in-the-loop” model, insisting that dental practitioners retain final diagnostic authority, with moderate concerns regarding data security and technical malfunction indicating that trust in AI remains conditional. Among dental practitioners, while an association was observed between formal AI training and more positive dental practitioner perceptions, this exploratory result does not constitute a proven predictor of adoption. However, perceptions of organizational support for AI implementation remained modest, revealing a critical gap between individual willingness and institutional readiness. These findings suggest that successful AI integration in dentistry requires a balanced approach combining clinician training, patient-centered communication, and well-established institutional frameworks.

## Data Availability

The datasets for this study are not publicly available to protect participant privacy, but anonymized data may be made available by the corresponding author upon reasonable academic request.
